# Ultrasound Findings in a Neonate During Anaphylactic Reaction to Total Parenteral Nutrition: A Case Report

**DOI:** 10.7759/cureus.33491

**Published:** 2023-01-07

**Authors:** Linah H Zaman, Haroon A Javaid, Nader Ashraf, Safwan Abbasi, Usama I Othman, Samira M Ghallab, Nabil Shehata

**Affiliations:** 1 College of Medicine, Alfaisal University College of Medicine, Riyadh, SAU; 2 Radiology, Saudi German Hospitals Group, Riyadh, SAU; 3 Pediatrics, Saudi German Hospitals Group, Riyadh, SAU; 4 Pediatrics and Neonatology, Saudi German Hospitals Group, Riyadh, SAU

**Keywords:** anaphylaxis, neonatal intensive care unit (nicu), neonate, total parenteral nutrition (tpn), ultrasound (u/s)

## Abstract

Total parenteral nutrition (TPN) is the intravenous delivery of nutrients and is commonly used in the Neonatal Intensive Care Unit (NICU). Hypersensitivity reactions to parenteral nutrition have seldom been described in the literature. Anaphylaxis is a potentially life-threatening emergency condition that can progress rapidly and involves multiple organ systems. We report a case of anaphylaxis due to TPN in a neonate with observed ultrasound findings during the acute episode never reported in the literature before.

## Introduction

Total parenteral nutrition (TPN) is the intravenous delivery of nutrients and is commonly used in the Neonatal Intensive Care Unit (NICU). It may serve as a pivotal intervention for neonates who cannot tolerate enteral nutrition such as premature babies, those with congenital gastrointestinal tract (GIT) anomalies, critical illnesses, or during post-surgical care [1]. Hepatobiliary and infectious complications are the commonly reported sequelae of TPN use. However, hypersensitivity reactions to parenteral nutrition have seldom been described in the literature. Reactions have been reported to be as mild as urticarial rash to more severe ones such as anaphylaxis [2-5].

Anaphylaxis is a potentially life-threatening emergency condition that can progress rapidly and involves multiple organ systems. It usually occurs secondary to exposure to an allergen but can also be diagnosed with rapidly developing hives associated with respiratory symptoms, circulatory collapse, or clinical signs of end-organ dysfunction [6]. As anaphylaxis is a clinical diagnosis, routine laboratory and imaging are not indicated in its workup during the acute episode. We report a case of anaphylaxis due to TPN in a neonate with observed ultrasound findings during the acute episode that has never been reported in the literature before.

## Case presentation

A preterm baby boy born at 32 weeks of gestation with a birth weight of 1.4kg, as a product of in-vitro fertilization (IVF) was admitted to the NICU for further management of prematurity and respiratory distress syndrome (RDS). The baby was born to a G2P1 mother with a history of hypothyroidism and penicillin allergy. He was given surfactant, ventilated, and extubate on the first day of life and started on TPN (containing glucose, amino acids and calcium) along with ampicillin and gentamicin. Upon examination, the patient was vitally stable and doing well. On the next day, the baby developed rapidly progressive generalized skin mottling/ecchymosis all over the body, followed by apnea, desaturation (SpO_2_ of 40%) and bradycardia. Blood pressure remained within normal limits. Upon examination, air entry was good and equal bilaterally. Some limb movement and muscular tone were present along with mild to moderate abdominal distension. Capillary refill time was greater than four seconds. The patient had received a new bag of standard TPN (containing amino acids, glucose, lipid emulsions, vitamins, trace elements and electrolytes), 10 minutes before the incident, and a penicillin infusion had finished an hour before.

The baby was urgently intubated and received positive pressure ventilation (PPV). TPN was discontinued immediately. Three doses of IM epinephrine (10 micrograms/kg) were given 6-7 minutes apart along with normal saline boluses (reaching about 120 mL/kg). One dose of IV hydrocortisone (5mg/kg) was also given. Upon resuscitation, the color of the body gradually improved (starting from the trunk, followed by the face and extremities) along with the heart rate and oxygen saturation. The baby fully recovered within 90 minutes. Laboratory investigations showed Hb of 11 mg/dL, WBC count of 20x10^9^/L with 14.5% eosinophils, and platelet count of 104x10^9^/L. IgE was 0 IU/mL. CRP was negative. Blood gas showed significant acidosis, which resolved spontaneously within three hours after recovery from the attack. No bicarbonate was given (Table 1). A team of three doctors and three nurses was involved in the immediate management of the neonate. Following the anaphylaxis shock, nutrition was switched to expressed breast milk which was gradually increased to the full feed. TPN was not reintroduced again.

**Table 1:** Venous Blood Gas (VBG) readings

	During resuscitation	After 1 hour	After 3 hours
pH	6.8	7.2	7.36
PCO2 (mmHg)	53.3	17.3	40
HCO3- (mmol/L)	8.5	10.3	21
Base deficit (mmol/L)	-25	-19	-2.7

**Figure 1:**
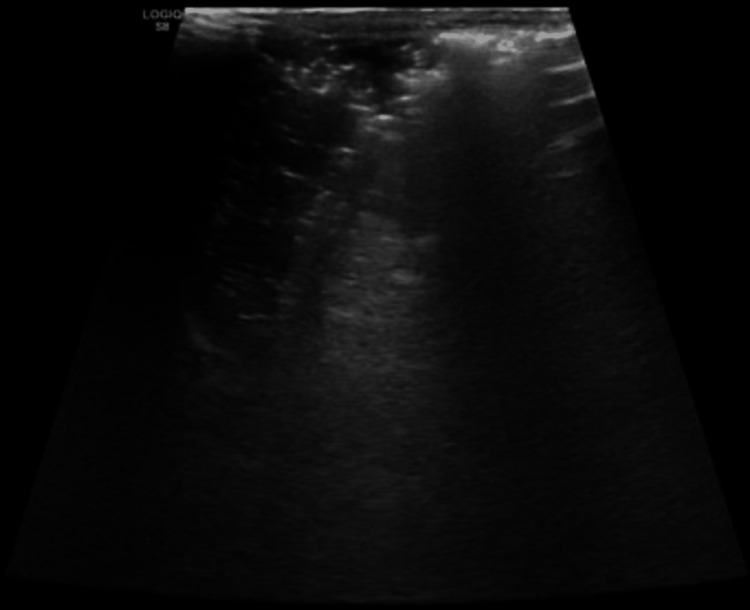
Abdominal ultrasound showing spleen normal in size with innumerable echogenic foci.

**Figure 2:**
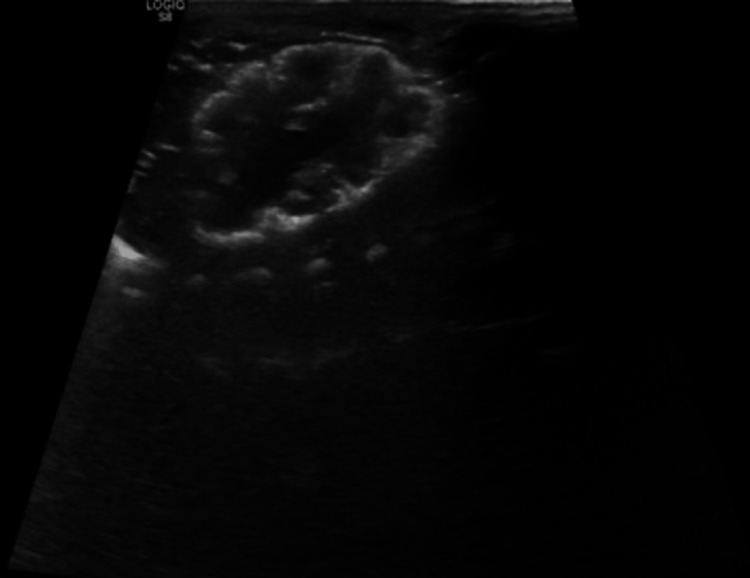
Abdominal ultrasound of right kidney showing loss of corticomedullary differentiation with minimal perinephric collection.

**Figure 3:**
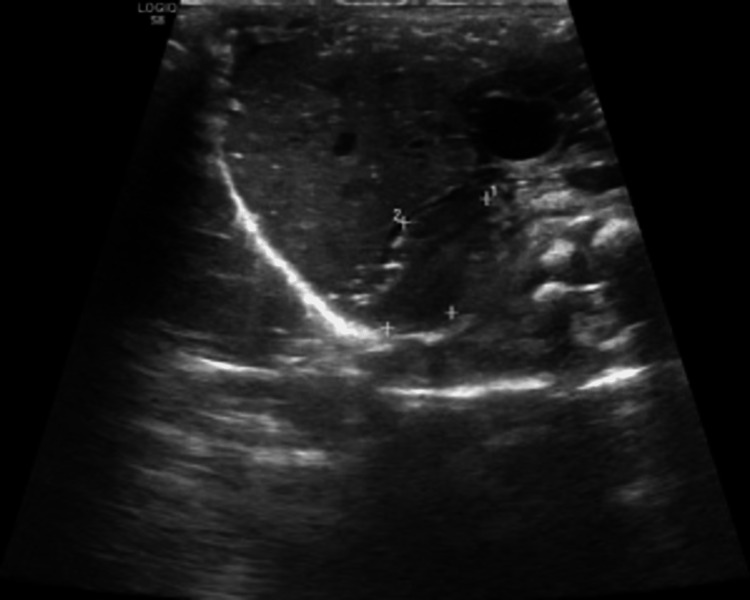
Abdominal ultrasound showing right suprarenal gland is enlarged (2.15 x 1.34 cm) with loss of corticomedullary differentiation.

**Figure 4:**
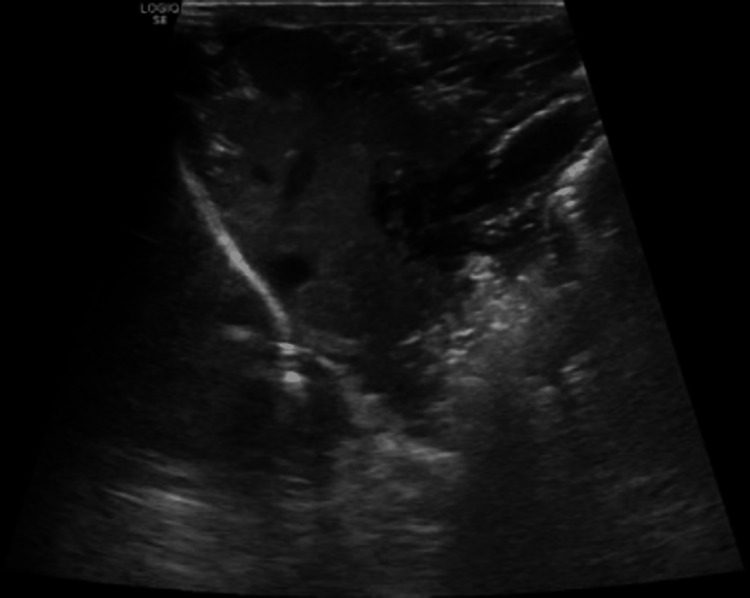
Abdominal ultrasound showing liver is normal in size. The presence of innumerable hypoechoic foci distributed in portal venous tracts and liver parenchyma. Periportal attenuation with nonvisualization of portal vein wall, collapsed IVC and attenuated hepatic vein also evident. Pericholecystic edema along with air in the gallbladder wall.

**Figure 5:**
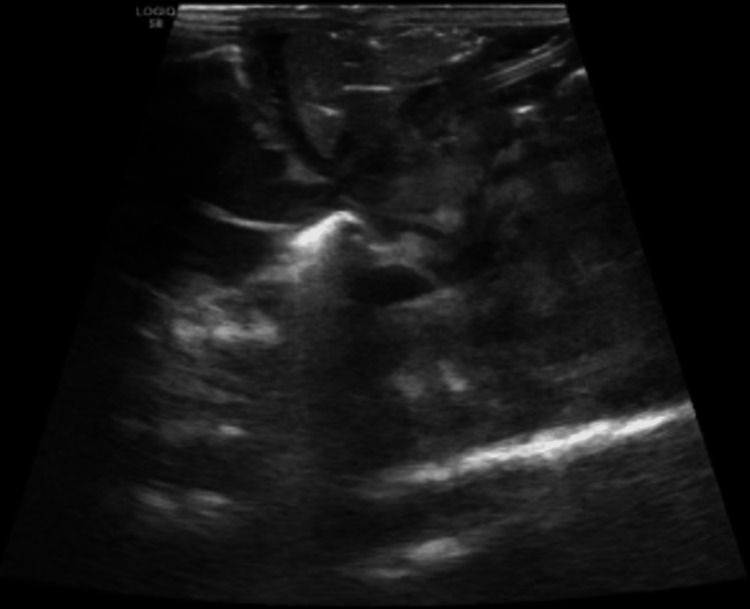
Abdominal ultrasound showing umbilical venous catheter (UVC) entering the left lobe of the liver, surrounded by edema.

**Figure 6:**
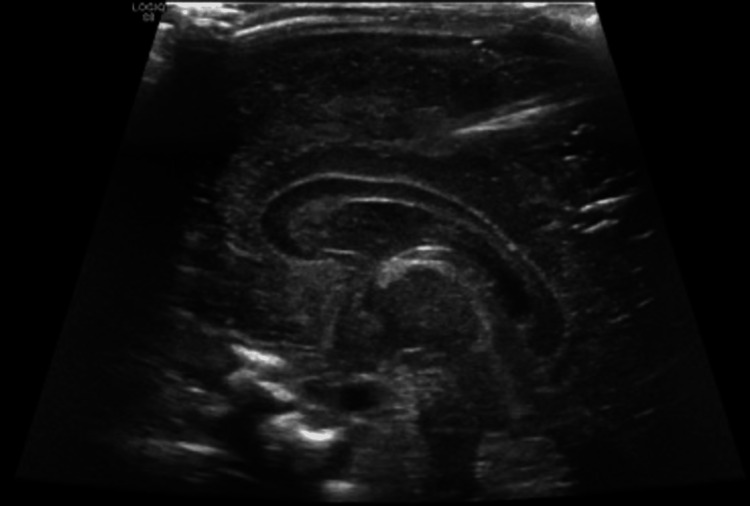
Transcranial ultrasound (sagittal view) showing diffuse hypoechoic appearance of the brain parenchyma with indistinct gray-white matter interface and symmetrical ventricular system attenuation. Scattered innumerable hypoechogenic foci. No signs of intracranial hemorrhage.

**Figure 7:**
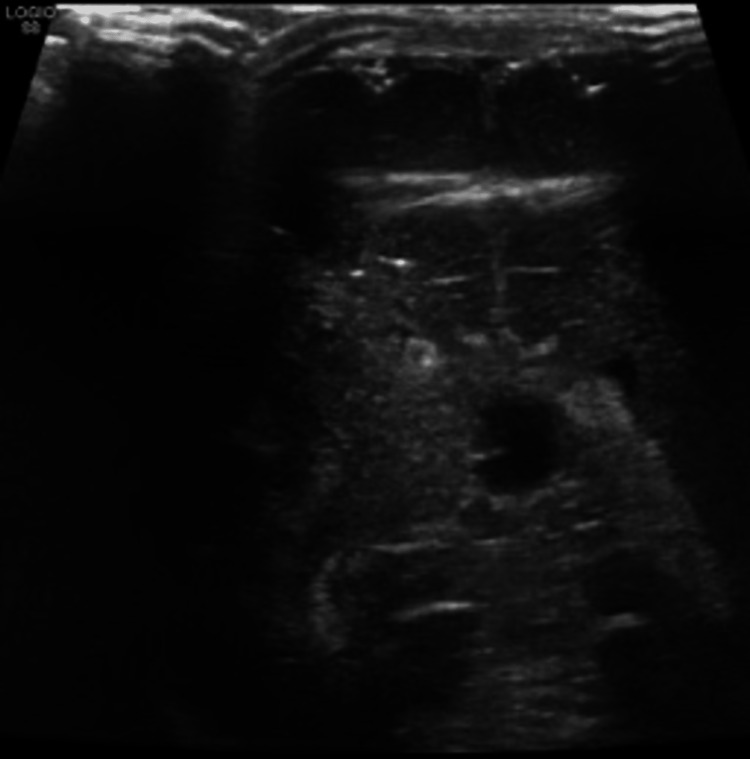
Transcranial ultrasound (transverse view) showing diffuse hypoechoic appearance of the brain parenchyma with indistinct gray-white matter interface and symmetrical ventricular system attenuation. Scattered innumerable hypoechogenic foci. No signs of intracranial hemorrhage.

**Figure 8:**
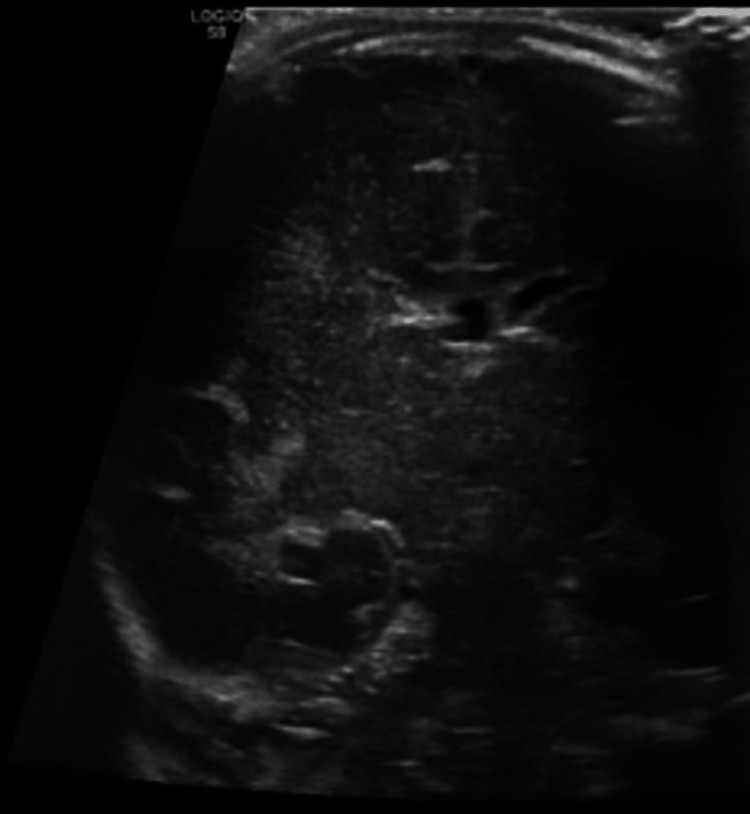
Transcranial ultrasound (20 minutes later) showing improvement in gray-white matter interface and the size of ventricles, decreased hypoechoic foci.

**Figure 9:**
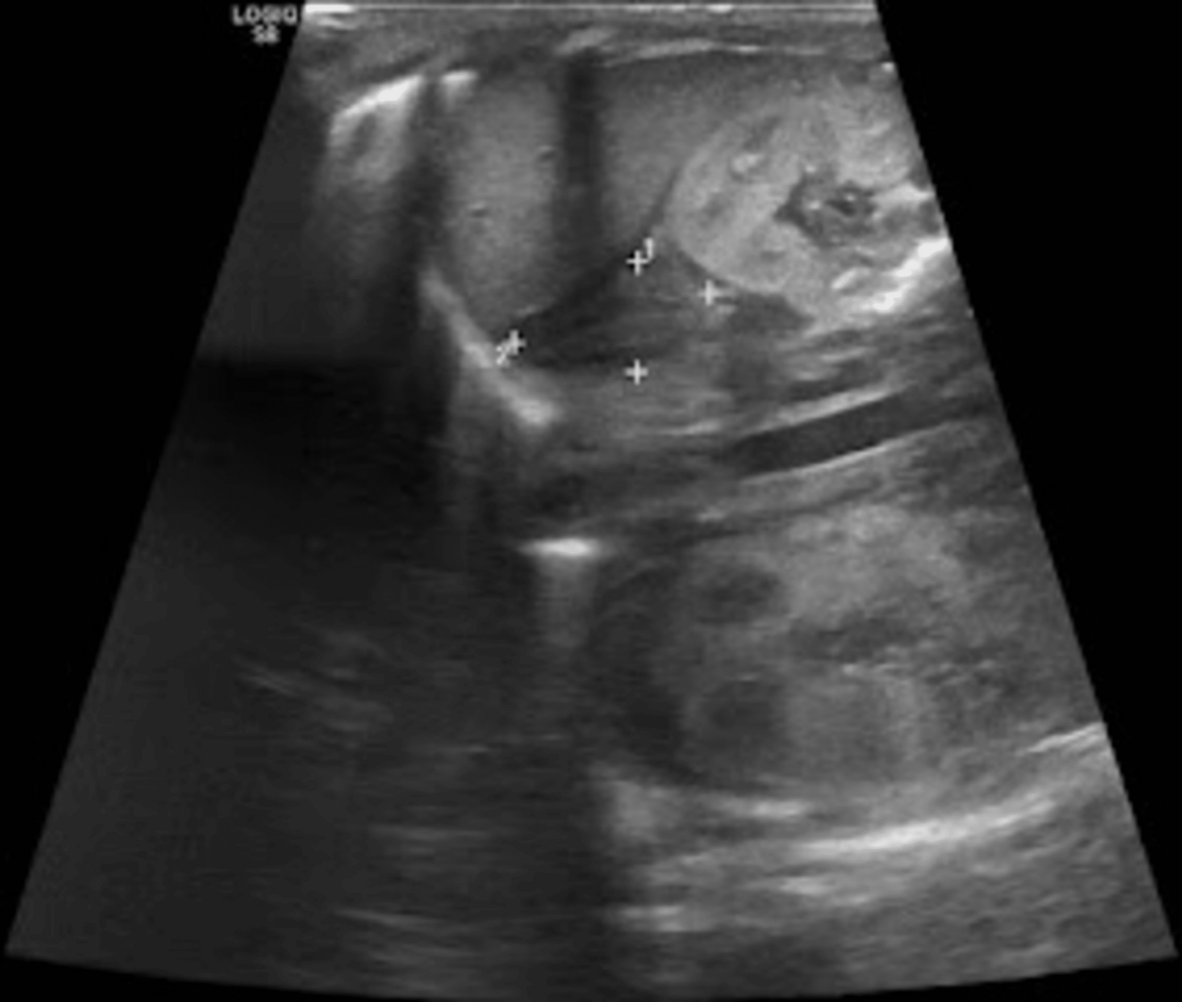
Abdominal ultrasound (10 minutes later) showing the size of right suprarenal gland decreased to 1.81 x 0.99 cm.

**Figure 10:**
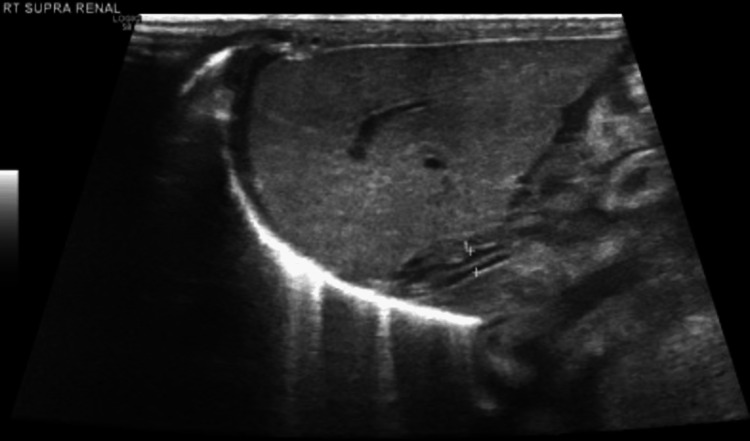
Abdominal ultrasound (20 minutes later) showing the size of right suprarenal gland decreased to normal.

Ultrasound of internal organs was incidentally performed during the anaphylactic attack. Transabdominally, the liver was normal in size and showed presence of innumerable hypoechoic foci distributed in portal venous tracts and liver parenchyma. Pericholecystic edema along with air in the gallbladder wall was observed. Spleen was normal in size with innumerable hypoechoic foci. Both kidneys demonstrated loss of corticomedullary differentiation with minimal perinephric collection. Suprarenal glands were bilaterally enlarged with loss of corticomedullary differentiation (Figures 1-5). Transcranial ultrasound showed diffuse hypoechoic appearance of the brain parenchyma with indistinct gray-white matter interface and symmetrical ventricular system attenuation as well as scattered innumerable hypoechogenic foci (Figures 6, 7). Mild ascites were also observed. A bedside echo done at the end of resuscitation demonstrated good contractility of the heart. Upon multiple follow-ups in the next 12 hours, all previous findings dramatically subsided with complete resolution back to normal (Figures 8-10).

## Discussion

We report the case of a preterm newborn who experienced severe symptoms of rapidly progressive skin mottling, bradycardia, apnea with desaturation, and mild abdominal distension on his second day of life, during his NICU stay. After initial management of intubation, normal saline boluses, epinephrine, and hydrocortisone, the symptoms improved within 90 minutes of onset. In the absence of clinical or laboratory evidence of sepsis, the constellation of symptoms with the fast onset following the start of TPN infusion and resolution after halting TPN clearly imply a hypersensitive response [7,8].

An antibiotic-induced anaphylactic reaction is a possible etiology for our case [9,10]. The timing of the symptoms coupled with the fact that the patient had safely received three doses of antibiotics prior to the current episode makes this possibility less likely. Some allergic reactions are documented to be from components in the method of delivery such as nickel (type of needle used), latex (TPN solution rubber stopper), or chlorohexidine gluconate (when used to disinfect skin) [11]. Latex-induced allergy is also unlikely as it usually involves infants who have undergone several surgeries or had mucosal exposure, such as urinary catheterization [12,13].

The multicomponent composition of TPN makes isolating the allergen involved challenging. Described components include both the therapeutically active and inactive constituents of parenteral nutrition solutions. Examples of the former described in the literature include the intravenous fat emulsion solution, multivitamin solution, and amino acid solution [11]. Inactive substances implicated in parenteral nutrition-related hypersensitivities include the emulsifiers, solubilizers, and wetting agents used in the formulation of such solutions [5]. Different formulas may contain other allergens such as egg yolk and soybean oil as components of lipid emulsions [3,14].

There is no precise diagnostic tool for determining the trigger of anaphylaxis in neonates, with documented cases pinpointing the exact component causing the allergy with a skin-prick test (SPT) being extremely rare as it is negative in the majority of patients [15]. Some cases make the diagnosis following the exclusion test and reintroduction of the various TPN components [16] while others have attempted to rule out possible allergens by Radioallergosorbent testing (RAST) [17]. For ethical reasons, the TPN reintroduction challenge was not attempted in our case. SPT and RAST were not carried out either, so the triggering component remains unknown. 

Most cases of anaphylaxis are IgE mediated. As the allergen binds to and cross-links IgE bound to immune cells such as mast cells and basophils, it activates them, leading to a release of a vast variety of substances that mediate the clinical manifestations of anaphylaxis. The chief mediator is histamine which accounts for the majority of the signs and symptoms in various organs, including the cutaneous (flushing, urticaria), respiratory (bronchospasm), and circulatory (hypotension, tachycardia) findings. Histamine acts on its respective receptors and may induce vasodilation, increased vascular permeability, and glandular secretion. Other implicated mediators included leukotrienes, cytokines, platelet-activating factors, bradykinin, and serotonin [18,19].

The diagnosis of anaphylaxis in the acute setting is a clinical one and radiological imaging is not indicated during the workup. In our case, ultrasound findings of the liver, gallbladder, kidney, spleen, and brain were incidentally observed during the anaphylactic episode. Interestingly, these changes started to resolve once the patient was stabilized and was completely reversed twelve hours after the event. To the best of our knowledge, such findings during an anaphylactic shock have never been reported in the literature before. These may contribute to understanding the complex pathophysiology of anaphylaxis, particularly in the context of the effects of mediators on different organs. Further research into this may result in the possibility of using imaging modalities to assist in the confirmation of a diagnosis of anaphylaxis.

## Conclusions

While anaphylaxis due to components of TPN is uncommon, there is a recent increase in reports of this complication. With the widespread use of TPN in the NICU, we want to highlight the possibility of fatal anaphylactic reactions in neonates. Further studies on ultrasound findings during anaphylaxis may expand our knowledge of these reactions in neonates and enhance the ability to recognize such reactions when they occur.
